# Effects of weight loss through dietary intervention on pain characteristics, functional mobility, and inflammation in adults with elevated adiposity

**DOI:** 10.3389/fnut.2024.1274356

**Published:** 2024-05-22

**Authors:** Susan J. Ward, Alison M. Coates, Sharayah Carter, Katherine L. Baldock, Carolyn Berryman, Tasha R. Stanton, Catherine Yandell, Jonathan D. Buckley, Sze-Yen Tan, Geraint B. Rogers, Alison M. Hill

**Affiliations:** ^1^Alliance for Research in Exercise, Nutrition and Activity (ARENA), University of South Australia, Adelaide, SA, Australia; ^2^Clinical and Health Sciences, University of South Australia, Adelaide, SA, Australia; ^3^Allied Health and Human Performance, University of South Australia, Adelaide, SA, Australia; ^4^Innovation, IMPlementation and Clinical Translation (IIMPACT), University of South Australia, Adelaide, SA, Australia; ^5^Persistent Pain Research Group, Hopwood Centre for Neurobiology, South Australian Health and Medical Research Institute (SAHMRI), Adelaide, SA, Australia; ^6^Institute for Physical Activity and Nutrition (IPAN), Deakin University, Burwood, VIC, Australia; ^7^Microbiome and Host Health, South Australian Health and Medical Research Institute (SAHMRI), Adelaide, SA, Australia

**Keywords:** diet, energy restriction, chronic pain, musculoskeletal pain, inflammation, overweight, obesity

## Abstract

**Background:**

The relationship between adiposity and pain is complex. Excess weight increases the risk for chronic musculoskeletal pain (CMP), driven by increased biomechanical load and low-grade systemic inflammation. Pain limits physical function, impacting energy balance contributing to weight gain. The primary aims of this study were to profile pain characteristics in participants with overweight or obesity and determine if weight loss through dietary-induced energy restriction, and presence of CMP, or magnitude of weight loss, was associated with changes in adiposity, pain, functional mobility, and inflammation.

**Methods:**

This was a secondary analysis of data from adults (25–65 years) with overweight or obesity (BMI 27.5–34.9 kg/m^2^) enrolled in a 3-month, 30% energy-restricted dietary intervention to induce weight loss (January 2019–March 2021). Anthropometric measures (weight, waist circumference and fat mass), pain prevalence, pain severity (McGill Pain Questionnaire, MPQ), pain intensity (Visual Analog Scale, VAS), functional mobility (timed up and go, TUG) and inflammation (high sensitivity C-Reactive Protein, hsCRP) were assessed at baseline and 3-months.

**Results:**

One hundred and ten participants completed the intervention and had weight and pain assessed at both baseline and 3-months. Participants lost 7.0 ± 0.3 kg, representing 7.9% ± 3.7% of body mass. At 3-months, functional mobility improved (TUG −0.2 ± 0.1 s, 95% CI −0.3, −0.1), but there was no change in hsCRP. Compared to baseline, fewer participants reported CMP at 3-months (*n* = 56, 51% to *n* = 27, 25%, *p* < 0.001) and presence of multisite pain decreased from 22.7% to 10.9% (*p* < 0.001). Improvements in anthropometric measures and functional mobility did not differ between those presenting with or without CMP at baseline. Improvements in pain were not related to the magnitude of weight loss.

**Conclusion:**

Weight loss was effective in reducing pain prevalence and improving functional mobility, emphasizing the importance of considering weight-loss as a key component of pain management.

**Clinical trial registration:**

identifier, ACTRN12618001861246.

## 1 Introduction

Chronic musculoskeletal pain (CMP) is the leading contributor to the burden of disease and disability worldwide, affecting 20%–33% of the world’s population (1). The body of evidence for the coexistence of obesity and CMP is growing (2, 3), with both obesity and CMP adversely impacting an individual’s health, functioning, and quality of life (3–5).

The prevalence of pain complaints rises incrementally across body mass index (BMI) categories (6–8) and with longer exposure to excess weight (9). To date, research has focused primarily on the relationship between BMI status and chronic pain, rather than adiposity (body fatness) (10). However, several population studies have documented that higher body fat mass is associated with musculoskeletal pain (9, 11), and is a risk factor for the development of chronic widespread, multi-site and site-specific (lower back, foot, and knee) pain (10, 12–17). Adiposity is of interest because it may be associated with increased systemic inflammation, and there is emerging evidence that inflammation may promote the progression of acute to chronic pain by altering the neurophysiological properties of peripheral pathways and central neurons of the pain system (neuroinflammation), contributing to central sensitization, and pain hypersensitivity (18–21).

Various studies have sought to evaluate the relationship between weight, inflammation and pain through mediation analyses or interventions designed to reduce body weight. Some studies suggest inflammation may mediate the link between higher body mass and osteoarthritis (OA) (22–24), while others have found no clear evidence that inflammatory factors influence pain (25), indicating a more direct impact of weight status on pain (26). However, an earlier meta-regression analysis of 11 randomized-controlled trials by Cooper et al. (27) failed to establish a significant relationship between pain reduction and weight loss in patients with musculoskeletal conditions. Nevertheless, the effectiveness of weight-loss interventions (diet and/or exercise) for reducing chronic (non-cancer) pain was recently recognized in a report by the Canadian Agency for Drugs and Technologies which evaluated three systematic reviews (specific to knee osteoarthritis) and two non-randomized studies (from weight management services) (28). More recent systematic reviews (totaling 33 studies) have also reported favorable effects of weight loss on pain and disability in CMP populations, however the breadth of pain measures used in these analyses were limited, and quality of included studies was low (29, 30). These reviews have predominantly included studies that focused on the link between obesity and excess mechanical load on weight bearing joints (knee and back) (27–30). Many of these studies included a physical activity or exercise component, precluding the ability to isolate changes in pain and function due to dietary-induced weight loss. Further, previous studies have been conducted predominantly in people with a pre-existing pain diagnosis, but it is common for people with overweight/obesity to have undiagnosed pain.

Therefore, the primary aims of this study were to: (1) profile sociodemographic and pain characteristics in free-living participants enrolled in a dietary intervention weight-loss trial, (2) determine if weight loss through dietary-induced energy-restriction, and presence of CMP, is associated with changes in weight, adiposity, functional mobility, inflammation, and pain, and (3) determine if magnitude of weight loss (clinically significant ≥ 5% weight loss or not) is associated with adiposity, pain, functional mobility, and inflammation. An exploratory aim was to quantify pair-wise change relationships between primary analysis outcome variables (adiposity, inflammation, and pain).

## 2 Materials and methods

### 2.1 Participants and study design

This was a secondary analysis of data from a dietary intervention trial conducted at the University of South Australia between January 2019 and March 2021. A protocol paper detailing the primary study design, participant eligibility criteria and details of outcomes has been published (31).

In brief, men and women from Adelaide and surrounding areas, aged 25–65 years with overweight and obesity (BMI 27.5–34.9 kg/m^2^) were recruited for a 9-month randomized controlled parallel arm intervention study. Participants were required to be non-smokers, consuming ≤14 standard alcohol drinks per week, not taking supplements or medication that might have potentially interfered with study measurements, and free from chronic health conditions such as diabetes, cardiovascular disease, thyroid, kidney or liver disease, or gastrointestinal disorders. Eligible participants provided written informed consent at screening visits. Ethical approval for the primary study was obtained from the University of South Australia Human Research Ethics Committee (Application ID: 201436) and was registered on the Australian New Zealand Clinical Trials Registry (ACTRN12618001861246).

### 2.2 Exposure: weight loss through dietary-induced energy-restriction

The dietary intervention was designed to investigate the effects of different snack foods (15% of energy from almonds or carbohydrate-rich snack foods) within a 30% energy-restricted diet on weight loss (3-months) and weight maintenance (6-months) (31). The effect of the different diets on the primary outcomes has been published (32). However, for this study, diet groups were combined as there were no differences in weight loss between groups (32), and we were interested in the overall effect of weight loss at 3-months (through energy restriction) on adiposity, pain, functional mobility, and inflammation. Weight loss was characterized as a continuous variable for assessing baseline and 3-month differences, and as a binary variable for assessing the magnitude of weight loss (<5% or ≥ 5% of baseline body weight over 3 months).

### 2.3 Exposure: chronic musculoskeletal pain

Body charts captured the site of pain experienced in the previous 24 h. Pain duration > 3 months at any site was used to define CMP, and considered as a binary variable to assess the effect of CMP presence.

### 2.4 Data collection

Participants attended the Clinical Trials Facility at the University of South Australia for screening, baseline, and 3-month assessments. Anthropometric measures [weight, height, waist circumference (WC), body composition], dietary intake, pain, functional mobility, and inflammation were assessed at baseline and 3 months. Prior to clinic assessments, participants were required to be fasted (>10 h) and have refrained from alcohol (>24 h). South Australian Government enforced Covid-19 restrictions interrupted 3-month clinic visits between April–June 2020. During this period participants measured their body mass at home using Bluetooth-enabled scales (Withings/Nokia WBS06, Nokia), but other anthropometric data (WC, body composition), blood samples, and functional mobility measures were not obtained. Weight captured by Bluetooth-enabled scales were used in analyses after determining there was no difference in the magnitude of weight loss achieved by participants assessed using Bluetooth-enabled scales compared to those whose weight was measured in the Clinical Trial Facility.

#### 2.4.1 Outcome measures

##### 2.4.1.1 Anthropometry

Height and weight measures were used to determine BMI [weight (kg)/height (m)^2^], classified according to WHO criteria (33). Waist circumference measurements were conducted as per the International Society for the Advancement of Kinanthropometry protocol (34). Whole-body dual-energy X-ray absorptiometry (DEXA) scans (Lunar Prodigy Model, GE Healthcare, Madison, Wisconsin, United States) were used to determine body composition (fat and lean mass, g, %) using enCORE 2015 software (V.13.31).

##### 2.4.1.2 Pain

Body charts captured the location of pain experienced in the previous 24 h (pain prevalence). In those reporting pain, the number of pain sites were recorded, along with pain duration. Participants also ranked pain sites from most troublesome to least troublesome. In participants reporting CMP, the Short Form McGill Pain Questionnaire (MPQ) measured the nature and severity of pain experienced at each pain site through 11 sensory and 4 affective words (35). Participants ranked each word on a Likert intensity scale from 0 (none), 1 (mild), 2 (moderate) to 3 (severe), to provide a total pain severity score out of 45. Current pain level at the site ranked most troublesome was captured via visual analog scale (VAS) in a subset of participants (*n* = 48, as VAS assessment began after study commencement). Participants selected a point along a 10 cm VAS from 0 representing ‘no pain’ and 10 ‘worst possible pain’, accordingly a higher score indicated greater pain intensity. The MPQ and VAS are suitable for the evaluation of pain complaints, and to measure the effects of interventions or pain relief in individuals, with both pain measures shown to be acceptable, reliable, and valid in adults (36).

The following measures were used to assess the effect of weight loss: pain prevalence, changes in number of pain sites, changes in the nature and severity of pain (MPQ), and changes in pain intensity (VAS). As pain locations were ranked from most to least troublesome, we determined pain severity and intensity scores from the pain site that was identified as the most troublesome at each time point. Sites of pain were additionally matched to the same location to gauge changes at the same location (for instance, comparing shoulder pain) for both time points. To calculate differences in pain intensity and severity from baseline, we assigned a score of 0 to any instance where CMP was absent at 3-months. If acute pain was reported at the 3-month time point it was not scored (and change scores were not determined).

##### 2.4.1.3 Functional mobility

Functional mobility was assessed by the timed up and go (TUG) test. Participants were timed getting up from a seated position in a chair, walking 3 meters, turning, returning to the chair, and sitting down again (37). Four trials were attempted with the average time (seconds) of the final three scored.

##### 2.4.1.4 Inflammatory markers

Following an overnight fast (>10 h), venous blood samples were collected into serum separator gel tubes. Samples were immediately transferred to a local pathology laboratory where high-sensitivity C-reactive protein [hsCRP (mg/L)] was measured. To account for acute inflammatory processes or infection, cut offs for high hsCRP were applied and values >10 mg/L excluded (38).

#### 2.4.2 Other assessments

Screening questionnaires captured age, gender, medication and supplement use, and socio-economic status (Socio-Economic Indices for Areas (SEIFA) deciles of advantage and disadvantage) (39). The diagnoses of pain provoking condition(s), analgesia usage, and past injuries and/or surgeries were documented for people with CMP.

Dietary intake was assessed with 4-day weighed food records captured in the week preceding baseline and 3-month visits. Weighed food records were analyzed using FoodWorks Nutritional Analysis Software Version 10 (Xyris Software, Brisbane) to provide estimates of daily energy intake. To exclude participants suspected of under or overestimating daily intake, established cut-offs of <2,090 kJ or >16,720 kJ/day (500–4,000 kcal) were applied to total energy intake (40). The dietary data for one participant was excluded due to kJ intake falling below the lower cut-off, which reduced the sample size to 135 for energy intake (Table 1).

**Table 1:** Baseline characteristics of participants by CMP status.

	All participants	Participants with CMP	Participants without CMP	*p*-value
	*n* = 136	*n* = 64	*n* = 72	
Women: Men (*n*, %)^*^	96 (71): 40 (29)	48 (35): 16 (12)	48 (35): 24 (18)	0.287
Age (years)	47.7 ± 10.7	48.7 ± 10.8	46.7 ± 10.7	0.277
SEIFA (0, disadvantage-10, advantage)^†‡^	7.0 ± 3.0	7.5 ± 4.0	7.0 ± 3.0	0.528
Medications, *n* (%)^*^				0.060
None	69 (50.7)	27 (42.2)	42 (58.3)	
Lipid lowering	8 (5.9)	4 (6.3)	4 (5.6)	
Antihypertensive	11 (8.1)	6 (9.4)	5 (6.9)	
Anti-anxiety/depression	18 (13.2)	11 (17.2)	7 (9.7)	
Hormone replacement	11 (8.1)	5 (7.8)	6 (8.3)	
Analgesic	13 (9.6)	8 (12.5)	5 (6.9)	
Reflux agents	7 (5.1)	5 (7.8)	2 (2.8)	
Other (contraceptive etc.)	29 (21.3)	16 (25.0)	13 (18.1)	
Supplements, *n* (%)^*^				0.603
None	100 (73.5)	47 (73.4)	53 (73.6)	
Multi/single vitamin/mineral	21 (15.4)	9 (14.1)	12 (16.7)	
Omega-3 Fatty acid	11 (8.1)	7 (10.9)	4 (5.6)	
Probiotic	1 (0.7)	0 (0.0)	1 (1.4)	
Calcium/Vitamin D	14 (10.3)	8 (12.5)	6 (8.3)	
Other (curcumin, glucosamine)	6 (4.4)	4 (6.3)	2 (2.8)	
Energy Intake (kJ/day)^‡^	9070.3 ± 2097.8	9308.0 ± 1948.3	8856.0 ± 2215.9	0.213
Weight (kg)	87.9 ± 11.5	88.4 ± 11.3	87.5 ± 11.7	0.635
BMI (kg/m^2^)	30.7 ± 2.3	30.8 ± 2.3	30.6 ± 2.2	0.587
Waist Circumference (cm)	102.0 ± 9.2	102.4 ± 9.7	101.7 ± 8.8	0.694
Body composition (DEXA)				
Total Fat Mass (kg)	36.1 ± 6.5	36.4 ± 6.5	35.8 ± 6.6	0.628
Total Percent Fat Mass (%)	42.7 ± 6.2	42.9 ± 6.1	42.6 ± 6.4	0.826
Total Lean Mass (kg)	48.6 ± 9.2	48.7 ± 9.1	48.5 ± 9.3	0.909
Total Percent Lean Mass (%)	55.4 ± 5.9	55.3 ± 5.8	55.6 ± 6.0	0.799
Functional mobility TUG (sec)^†‡^	5.12 ± 1.01	5.18 ± 0.89	5.09 ± 1.10	0.246
Inflammation, hsCRP (mg/L)^†§^	1.85 ± 2.2	1.60 ± 1.90	2.05 ± 2.40	0.104
Pain reported, *n* (%)	89 (65.4)			
Pain not CMP	25 (18.4)			
CMP reported	64 (47.1)			
AIHW MSK classifications, *n* (%)^¶^				
No diagnosis		31 (50.8)		
Back pain/problems		9 (14.8)		
Osteo arthritis		9 (14.8)		
Rheumatoid arthritis		0 (0.0)		
Osteoporosis		0 (0.0)		
Other MSK condition (gout, soft tissue etc.)		12 (19.7)		
MSK fracture or surgery (past 5 years)		10 (16.4)		
Pain medication classification, *n* (%)^¶^				
No medication		35 (57.3)		
Paracetamol		11 (18.0)		
NSAID		18 (29.5)		
Opioid		1 (1.6)		
Antidepressant (for pain)		0 (0.0)		
Anticonvulsant		2 (3.3)		
Supplement (for pain)		1 (1.6)		
MPQ worst CMP site (0–45), (*n* = 64)		7.8 ± 6.3		
VAS worst CMP site (0–10), (*n* = 44)		4.0 ± 2.1		

Values are mean ± standard deviation for normally distributed data with independent t-test for differences between CMP and no CMP groups. ^*^Relative numbers (*n*, %) with chi-square test for difference. ^†^Median ± interquartile range for skewed data with Mann–Whitney U test for differences. ^**‡**^*n* = 135 (CMP *n* = 64, no CMP *n* = 71). ^§^hsCRP *n* = 122 (CMP *n* = 56, no CMP *n* = 66), missing data, *n* = 7, excluded data for levels ≥ 10 mg/L, *n* = 7. ^¶^Reported in participants with CMP only, missing data, *n* = 3. AIHW, Australian Institute of Health and Welfare; CMP, chronic musculoskeletal pain; hsCRP, high sensitivity C-reactive protein; DEXA, dual-energy X-ray absorptiometry; IQR, interquartile range; MPQ, McGill Pain Questionnaire; MSK, musculoskeletal; NSAID, non-steroidal anti-inflammatory drugs; SEIFA, Socio-Economic Indices for Areas; SD, standard deviation; TUG, timed up and go; VAS, visual analog scale.

## 3 Sample size

The sample size of this study was determined by the availability of weight and pain data in participants enrolled in the primary study (31, 32). Thus, 136 participants with weight and pain data at baseline determined the sample size for profiling sociodemographic and pain characteristics (primary aim 1). Participants with complete weight and pain data at both baseline and 3-months (*n* = 110) governed the sample size for investigating whether weight loss and presence of CMP, was associated with change in adiposity, functional mobility, inflammation, and pain (primary aims 2 and 3).

## 4 Statistical analysis

Statistical analyses were undertaken using SPSS version 28.0 (SPSS, Chicago, IL, United States). For all analyses, a statistical significance of *p* < 0.05 was set.

### 4.1 Sociodemographic and pain characteristics

Participant demographics and pain characteristics were documented for all participants with weight and pain data at baseline. Sample means and standard deviations (SD), and medians and interquartile ranges (IQR) for continuous variables, and counts and proportions, calculated for discrete variables, were descriptively reported overall, and for those with and without CMP at baseline (i.e., reporting pain of ≥3 months duration or reporting no pain/pain of <3 months duration, respectively). Following normality testing using histogram plots and Shapiro–Wilk test, differences between those with and without CMP at baseline were assessed using independent t-test and Mann Whitney-U tests as appropriate. Chi-square test for homogeneity was used to test the difference between proportion of men and women reporting CMP at baseline.

### 4.2 Effect of weight loss and presence of CMP on anthropometry, functional mobility, inflammation, and pain

Pre-post-intervention analyses were conducted in participants with complete weight and pain data (pain prevalence) at baseline and 3-months. This excluded seven participants who reported CMP at 3-months that was not present at baseline because it led to uncertainty about the presence of CMP. McNemar’s test was used to determine if there was a difference in the proportion of participants reporting pain at baseline vs. 3-months (pain prevalence). Effects of weight loss through dietary-induced energy-restriction (time effect) on outcomes (anthropometry, functional mobility, and inflammation), and differences in outcomes between those with and without CMP at baseline (group effect), were determined using mixed effects models, with time and group as fixed effects, group by time as an interaction, and participant random intercepts to account for repeated measures. Covariate importance was assessed by Akaike information criterion (AIC) and Bayesian information criterion (BIC) values. Estimated marginal means (EMM) and standard error of the mean (SEM) are reported for the mixed effects models.

A mixed model including time as a fixed effect and participant random intercepts to account for repeated measures was used to evaluate changes in pain severity and intensity over time in the CMP group only. AIC and BIC values determined covariate inclusion in this model. McNemar’s test was used to test the differences in proportions reporting pain at the different sites in the body.

### 4.3 Effect of magnitude of weight loss on adiposity, functional mobility, inflammation, and pain

We evaluated whether changes in outcomes (body composition, functional mobility, inflammation, and pain) after weight loss through dietary-induced energy-restriction (time effect) differed between those who achieved weight loss considered clinically meaningful due to associations with improved health outcomes, compared to those who did not reach this level of weight loss (group effect; i.e., ≥ 5% weight loss and <5% weight loss) (41). The same model structure and covariate selection as described in 4.2 was used, except with the magnitude of weight loss as the fixed group effect of interest. Where a group by time interaction was identified, Bonferroni *post hoc* tests were applied.

#### 4.3.1 Exploratory associations

Regression analyses were conducted in (1) participants completing the intervention to understand the relationship between changes in adiposity and changes in inflammation (hsCRP; *n* = 70–75), and (2) in those with CMP at baseline, to determine the relationship between change in inflammation and change in pain characteristics (*n* = 19–39). Age, gender, and baseline BMI were included in the linear regression models.

## 5 Results

**Figure 1:**
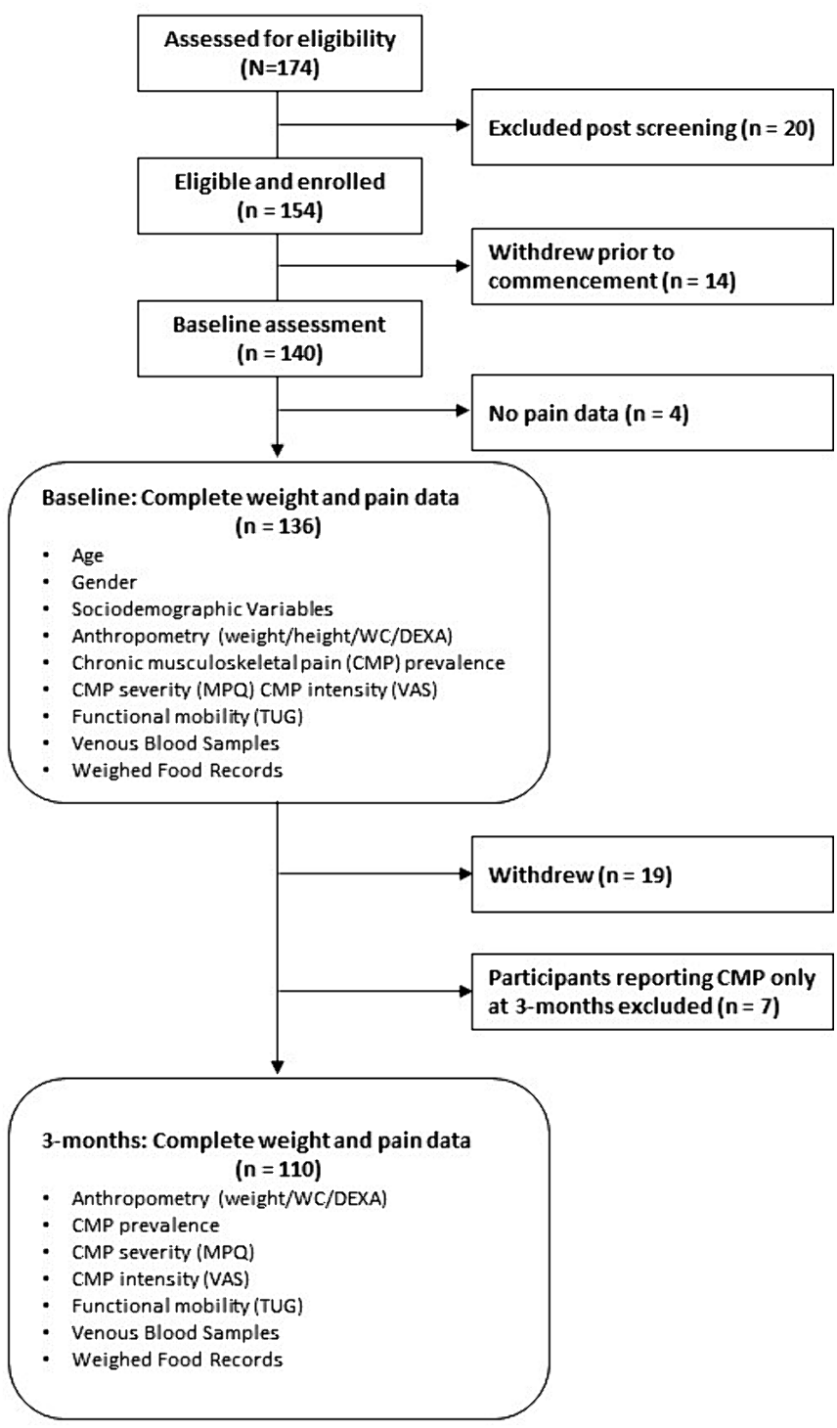
Flow of participants through the study and sample sizes for analyses. BMI, body mass index; CMP, chronic musculoskeletal pain; DEXA, dual-energy X-ray absorptiometry; MPQ, McGill Pain Questionnaire; SES, socio-economic status; TUG, timed up and go; VAS, visual analog scale; WC, waist circumference.

Of 174 people assessed for eligibility, 140 participants completed baseline assessments with both weight and pain (prevalence) data obtained from 136 participants at baseline, and 110 participants had weight and pain data at both baseline and 3-months (Figure 1).

Baseline characteristics of participants with weight and pain data (*n* = 136) are presented in Table 1. Most participants were women (71%), body weight ranged from 64.5–118.7 kg, and 62.5% of participants fell into the obese BMI category (mean ± SD; 30.7 ± 2.3 kg/m^2^). Nearly half (47%) of participants enrolled in the study reported CMP at baseline. Considering participants with or without CMP at baseline, there were no differences in age, gender, SEIFA, use of medications or supplements, energy intake (kJ/day), anthropometric measurements (weight, WC, or body composition), functional mobility (TUG), or inflammation (hsCRP) (Table 1).

In participants with CMP, most reported non-specific chronic pain (*n* = 31, 50.8%), while similar numbers reported a diagnosis of osteoarthritis (*n* = 9, 14.8%) or non-specific back pain (*n* = 9, 14.8%), and other musculoskeletal conditions (capturing soft tissue injuries, gout etc.) was reported in 12 (19.7%). The use of pain medication for CMP was reported in 43 and 16% reported a musculoskeletal fracture or surgical procedure in the previous 5 years (Table 1).

### 5.1 Impact of weight loss and presence of CMP

By 3-months, all participants (*n* = 110) reduced their total daily energy intake (mean change ± SEM −3,250 ± 171 kJ/day, 95% CI −3,588, −2,911) with accompanying reductions in weight (−7.0 ± 0.3 kg, 95% CI −7.6, −6.3), WC (−7.2 ± 0.5 cm, 95% CI −8.2, −6.1), percent fat mass (−3.9 ± 0.2, 95% CI −4.3, −3.4), and an increase in percent lean mass (3.5 ± 0.2, 95% CI 3.1, 3.9). Table 2 presents the effect of weight loss (time) and the interaction with presence of CMP (group by time). See Supplementary Table S1 for baseline characteristics of this sub-group. Models were adjusted for age, gender, SEIFA (tertiles), analgesic medication use, and anti-inflammatory/analgesic supplement use. Baseline BMI was also included for non-weight outcomes (AIC and BIC values presented in Supplementary Table S2). A significant improvement in functional mobility was observed (TUG change −0.2 ± 0.1 s, 95% CI −0.3, −0.1) but there was no change in levels of circulating hsCRP (−0.1 ± 0.2 mg/L, 95% CI −0.5, 0.3). At 3-months, CMP prevalence reduced from 50.9% to 24.6% (*p* < 0.001) and multisite pain (CMP in two or more sites) decreased from 22.7% to 10.9% (*p* < 0.001). There were no group by time interactions indicating that the presence of CMP did not influence the anthropometric, physical functioning (TUG) or inflammation (hsCRP) outcomes.

**Table 2:** Model estimated marginal means (± standard error) and mean difference (95% CI) for weight, adiposity, functional mobility, and inflammation by presence of CMP over time.

	CMP			No CMP			*p*-value		
	Baseline	3-months	Mean difference (95% CI)	Baseline	3-months	Mean difference (95% CI)	Group	Time	Group × time
Weight (kg)	*n* = 56	*n* = 56		*n* = 53	*n* = 53				
	89.0 ± 1.3	82.1 ± 1.3	−6.8 (−7.8, −5.9)	87.0 ± 1.3	79.9 ± 1.3	−7.1 (−8.1, −6.1)	0.235	<0.001	0.700
BMI (kg/m^2^)	30.9 ± 0.3	28.5 ± 0.3	−2.4 (−2.7, −2.1)	30.5 ± 0.3	28.0 ± 0.3	−2.5 (−2.8, −2.1)	0.367	<0.001	0.748
WC (cm)	*n* = 56	*n* = 46		*n* = 53	*n* = 44				
	102.9 ± 1.1	96.1 ± 1.1	−6.8 (−8.2, −5.3)	101.5 ± 1.1	93.9 ± 1.2	−7.6 (−9.1, −6.1)	0.249	<0.001	0.427
DEXA	*n* = 56	*n* = 44		*n* = 53	*n* = 41				
Fat mass (kg)	36.4 ± 0.9	30.9 ± 0.9	−5.5 (−6.4, −4.6)	35.7 ± 0.9	29.7 ± 0.9	−6.0 (−6.9, −5.1)	0.449	<0.001	0.409
Percent fat mass (%)	42.5 ± 0.6	38.8 ± 0.6	−3.7 (−4.3, −3.0)	42.7 ± 0.6	38.6 ± 0.6	−4.1 (−4.8, −3.5)	0.958	<0.001	0.302
Lean mass (kg)	49.4 ± 0.7	48.4 ± 0.7	−1.0 (−1.3, −0.6)	48.3 ± 0.7	47.3 ± 0.7	−1.1 (−1.5, −0.7)	0.241	<0.001	0.678
Percent lean mass (%)	55.6 ± 0.6	59.0 ± 0.6	3.3 (2.8, 3.9)	55.5 ± 0.6	59.2 ± 0.6	3.7 (3.1, 4.3)	0.932	<0.001	0.394
Functional mobility, TUG (sec)	*n* = 56	*n* = 45		*n* = 52	*n* = 44				
	5.3 ± 0.1	5.1 ± 0.1	−0.2 (−0.4, −0.0)	5.3 ± 0.1	5.0 ± 0.1	−0.2 (−0.4, −0.0)	0.753	0.001	0.791
Inflammation, hsCRP (mg/L)	*n* = 49	*n* = 41		*n* = 49	*n* = 37				
	2.4 ± 0.3	2.4 ± 0.3	0.01 (−0.5, 0.5)	2.5 ± 0.3	2.2 ± 0.3	−0.2 (−0.8, 0.3)	0.925	0.562	0.517

All models adjusted for age, gender, SEIFA (tertiles), analgesic medication use (yes/no) and anti-inflammatory/analgesic supplement use (yes/no), plus baseline BMI for non-weight outcomes. BMI, body mass index; CMP, chronic musculoskeletal pain; CI, Confidence Interval; DEXA, dual-energy X-ray absorptiometry; hsCRP, high sensitivity C-reactive protein; TUG, timed up and go; WC, waist circumference.

**Figure 2:**
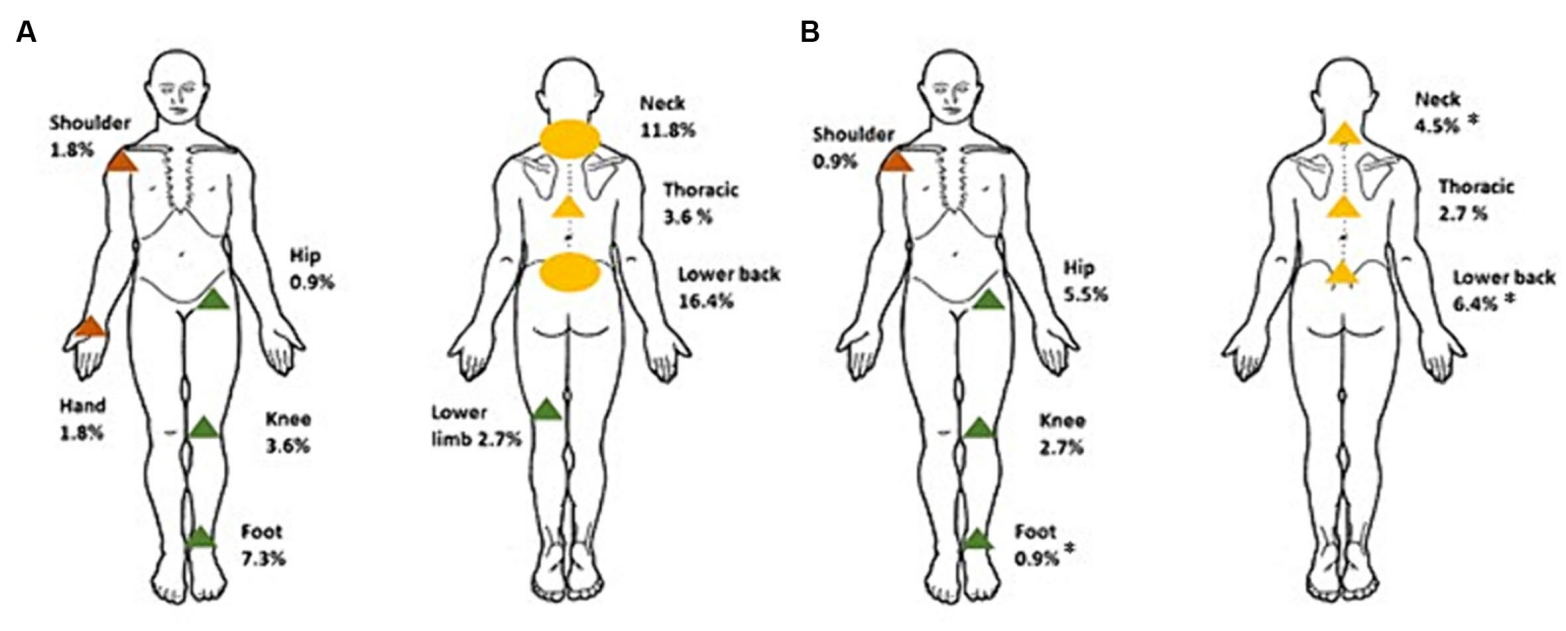
Distribution of most troublesome site of CMP reported in participants at baseline **(A)** and 3-months **(B)**. ^*^Significantly different to baseline, *p* < 0.05. Circles represent where >10% of participants reported CMP, and triangles where <10% of participants reported CMP. Green corresponds to pain complaints in the lower limb, orange, the upper limb, and yellow, spinal pain.

Changes in pain variables in participants with CMP following intervention are shown in Supplementary Table S3 (see Supplementary Table S2 for AIC and BIC values). There was a reduction in the average number of pain sites (mean change ± SEM, −0.8 ± 0.1, 95% CI −1.1, −0.6) by 3-months compared to baseline. There were significant reductions in pain severity (MPQ change −3.2 ± 0.9, 95% CI −4.8, −1.6) and pain intensity (VAS change −1.8 ± 0.5, 95% CI −2.8, −0.9) at the site of pain that participants identified as being most troublesome. When areas of pain were matched for site, there were again significant reductions in pain severity (MPQ change −3.1 ± 0.9, 95% CI −4.8, −1.4) and pain intensity (VAS change −2.6 ± 0.5 (26%), 95% CI −3.6, −1.5). A 20% change in VAS has been defined as a clinically significant reduction in pain intensity (42). Figure 2 provides a visual representation of the specific sites of CMP at baseline compared to 3-months. Collectively, spinal (neck, thoracic, lower back) pain reduced in prevalence from 31.8% in all participants to 13.6% (*p* < 0.001), upper limb (shoulder and hand) pain reduced from 3.6% to 0.9% (*p* = 0.250), and lower limb (hip, knee, foot) pain from 14.5% to 9.0% (*p* = 0.210). By sites, significant improvements were observed for some [lower back (*p* = 0.011), neck (*p* = 0.021) and foot (*p* = 0.016)], but not in all (no significant differences in thoracic, shoulder, hand, hip, or knee pain).

### 5.2 Impact of magnitude of weight loss

Clinically significant weight loss (≥5% of body weight) was achieved in 80% of participants, with an average 7.9 ± 3.7% reduction in body weight for all participants (*n* = 110). Changes in outcomes by weight loss categories (clinically significant weight loss ≥ 5% of body weight compared to <5% loss of body weight) showed a near significant group by time improvement in physical functioning (TUG; *p* = 0.055). However, magnitude of weight loss was not related to changes in inflammation (hsCRP) or any pain measures (Table 3). See Supplementary Table S2 for model AIC and BIC values.

**Table 3:** Model estimated marginal means (± standard error) and mean difference (95% CI) for adiposity (WC, body composition), functional mobility, and pain by magnitude of weight loss over time.

	WL > 5%			WL < 5%			*p*-value		
	Baseline	3-months	Mean difference (95% CI)	Baseline	3-Months	Mean difference (95% CI)	Group	Time	Group × time
WC (cm)	*n* = 88	*n* = 73		*n* = 21	*n* = 17				
	102.1 ± 0.9	93.6 ± 0.9	−8.5 (−9.5, −7.5)	102.8 ± 1.8	101.2 ± 1.8	−1.5 (−3.6, 0.5)	0.035	<0.001	<0.001
DEXA	*n* = 88	*n* = 70		*n* = 21	*n* = 15				
Fat mass (kg)	35.7 ± 0.7	29.1 ± 0.7	−6.6 (−7.1, −6.0)	37.3 ± 1.4	35.4 ± 1.4	−1.9 (−3.1, −0.7)	0.011	<0.001	<0.001
Percent fat mass (%)	42.3 ± 0.5	38.0 ± 0.5	−4.4 (−4.8, −4.0)	43.5 ± 1.0	42.1 ± 1.0	−1.5 (−2.4, −0.6)	0.016	<0.001	<0.001
Lean mass (kg)	48.9 ± 0.5	47.6 ± 0.5	−1.3 (−1.6, −1.1)	48.6 ± 1.1	49.0 ± 1.1	0.4 (−0.1, 1.0)	0.669	0.007	<0.001
Percent lean mass (%)	55.8 ± 0.4	59.8 ± 0.5	4.0 (3.6, 4.3)	54.6 ± 0.9	56.0 ± 0.9	1.4 (0.6, 2.2)	0.016	<0.001	<0.001
Functional mobility TUG (secs)	*n* = 87	*n* = 72		*n* = 21	*n* = 17				
	5.3 ± 0.1	5.0 ± 0.1	−0.3 (−0.4, −0.1)	5.4 ± 0.2	5.4 ± 0.2	0.04 (−0.2, 0.3)	0.218	0.153	0.055
Inflammation hsCRP (mg/L)	*n* = 78	*n* = 62		*n* = 20	*n* = 16				
	2.3 ± 0.2	2.1 ± 0.2	−0.2 (−0.6, 0.2)	2.9 ± 0.4	3.1 ± 0.5	0.2 (−0.6, 1.0)	0.082	0.976	0.412
Number of CMP sites	*n* = 44	*n* = 44		*n* = 12	*n* = 12				
	1.7 ± 0.1	0.9 ± 0.1	−0.8 (−1.1, −0.6)	1.3 ± 0.2	0.5 ± 0.2	−0.8 (−1.3, −0.3)	0.122	<0.001	0.979
MPQ worst CMP site (0–45)	*n* = 44	*n* = 38		*n* = 12	*n* = 11				
	8.4 ± 0.9	5.0 ± 0.9	−3.5 (−5.3, −1.7)	5.4 ± 1.7	3.3 ± 1.8	−2.1 (−5.5, 1.2)	0.179	0.005	0.481
VAS worst CMP site (0–10)^†^	*n* = 31	*n* = 32		*n* = 8	*n* = 9				
	4.3 ± 0.4	2.6 ± 0.4	−1.7 (−2.8, −0.6)	4.0 ± 0.8	1.8 ± 0.8	−2.2 (−4.3, −0.1)	0.427	0.002	0.652
MPQ matched CMP site (0–45)	*n* = 35	*n* = 35		*n* = 11	*n* = 11				
	7.7 ± 1.0	4.5 ± 1.0	−3.3 (−5.3, −1.3)	6.4 ± 1.8	3.7 ± 1.8	−2.7 (−6.3, 0.8)	0.581	0.005	0.795
VAS matched CMP site (0–10)^†^	*n* = 20	*n* = 20		*n* = 7	*n* = 7				
	4.2 ± 0.5	1.7 ± 0.5	−2.5 (−3.8, −1.3)	3.3 ± 0.8	0.6 ± 0.8	−2.7 (−4.8, −0.5)	0.181	<0.001	0.895

All models adjusted for age and gender, SEIFA (tertiles), analgesic medication use (yes/no) and anti-inflammatory/analgesic supplement use (yes/no), and baseline BMI for non-weight outcomes. ^†^Baseline BMI excluded due to model’s convergence and validity issues. BMI, body mass index; CMP, chronic musculoskeletal pain; CI, Confidence Interval; DEXA, dual-energy X-ray absorptiometry; hsCRP, high sensitivity C-reactive protein; MPQ, McGill Pain Questionnaire; SEIFA, Socio-Economic Indices for Areas; TUG, timed up and go; VAS, visual analog scale; WC, waist circumference; WL, weight loss.

### 5.3 Relationship between adiposity, inflammation, and pain

Changes in adiposity were associated with change in hsCRP (weight, β = 0.273, 95% CI 0.024, 0.244; WC, β = 0.240, 95% CI 0.003, 0.160; fat mass (kg), β = 0.263, 95% CI 0.00002, 0.0003; percent fat mass (%), β = 0.268, 95% CI 0.005, 0.104). In participants reporting CMP, there was a significant association between change in hsCRP and pain severity scores, but only when identical pain sites were compared between visits (Supplementary Table S4).

## 6 Discussion

This study investigated pain characteristics in adults with overweight and obesity participating in a dietary intervention for weight loss. In this population, baseline pain prevalence was high with pain determined to be chronic (extending beyond 3 months) in 47% of participants, and multisite (>1 site) pain present in 21% of participants (~45% of those presenting with CMP). Capturing sites of pain showed the distribution of pain was highest in areas of weight bearing (spine and lower limbs). The main finding was that participants achieved significant weight loss, resulting in improved prevalence, severity and intensity of CMP, and functional mobility.

Pain complaints were high in areas of mechanical load. The distribution of pain is not commonly reported, as interventions for weight loss are usually directed at specific pain populations, such as hip or knee osteoarthritis, rather than in community samples. Though a retrospective study of pain presentation in a weight-management service (mean BMI 50.8 ± 8.1 kg/m^2^) similarly described high pain prevalence with multisite pain reported in two-thirds of participants (43). As with previous studies, also performed in weight management services (43, 44), a reduction in pain prevalence in several weight bearing joints along with a reduction in multisite pain was seen with weight loss. The change in pain distribution with weight loss was varied, with a reduction in lower back, neck, and foot pain, and, although not reaching significance, hip pain complaints increased. Future studies should consider whether this shift in pain presentation is due to alterations in posture along with changes in mechanical loading associated with increased movement after weight loss.

The potential for weight loss to be impacted by the presence of severe pain prior to intervention has been investigated (45–47). A retrospective study of 386 adults in the United States, determined that joint pain was predictive of weight loss in women but not men (mean BMI 40.7 ± 10.12 kg/m^2^) (45), and in a weight-loss intervention in United States veterans (85% men, mean BMI 36.4 ± 6.2 kg/m^2^), those with coexisting severe pain and overweight/obesity lost significantly less weight than those reporting mild or moderate pain (46). Similarly, obese individuals (mean BMI 46.3 ± 7.2 kg/m^2^) reporting severe pain, upon entry to a weight management service, lost less weight than those reporting moderate to no pain (48). Furthermore, after similar initial weight loss in the first 6-months, weight loss at 2 years was less likely to be sustained in participants presenting with CMP, compared to those without, in a behavioral weight-loss program (47). However, we saw no difference in improvements in adiposity between pain free participants and those reporting CMP at baseline. These differences may be due to the BMI of enrolled participants in the aforementioned studies, which was above 35 kg/m^2^, the upper limit set for our study; pain may be a barrier to weight loss in those with higher BMIs where associated functional impairments and impacts to physical activity are also higher (45, 48).

Elevated levels of proinflammatory cytokines interleukin-6 (IL-6) and CRP have been linked to central sensitization and chronic pain, suggesting involvement in these processes (49, 50). These cytokines are indicative of low-grade inflammation in obesity, where IL-6 is produced by adipose tissue, and CRP (whose production is induced by Il-6) serves as a biomarker (51). Improvements in pain characteristics following weight loss could be the result of lowering of obesity mediated inflammation alongside the lessening of mechanical load (52–54). However, most participants (73% at baseline and 71% at 3-months) presented with hsCRP levels below 3 mg/L, considered within the normal range in previous dietary studies (55), and we saw a non-significant reduction in hsCRP in the no CMP group compared to a trivial rise in the CMP group post-intervention. Interestingly, despite the sample having low levels of inflammation at baseline, the exploratory regression analysis showed reductions in adiposity were associated with reductions in hsCRP. Furthermore, significant relationships between reductions in hsCRP levels and pain severity change scores were only observed for subgroups where identical pain sites were matched. It remains unclear as to what level of CRP is important in the association with CMP (56–58). While some studies suggest a mediating role for inflammatory biomarkers (particularly leptin) in the link between excess weight and OA (22–24), others have found no clear indirect effect through measured inflammatory parameters on back and hand pain suggesting direct mechanical loading effects have a greater influence (25, 26). Again, the limited BMI range for this study may also have impacted our ability to determine shifts in inflammatory status with weight loss.

Consistent with previous single-arm interventions (low energy diet, multi-disciplinary weight-loss program, weight-management services) reporting on chronic pain in people with overweight and obesity, we observed significant improvements in pain prevalence and intensity (43, 46, 47, 59, 60). Although a critical review, primarily of studies involving knee osteoarthritis, recognized the effectiveness of weight loss for chronic pain, the findings were varied for pain prevalence and pain severity, with favorable or null effects (28). Subsequent systematic reviews also reported limited and low-quality evidence to support weight-loss interventions for chronic osteoarthritis-related, and low back pain (29, 30), indicating a need for further high-quality interventions. Similar improvements in pain measures were observed in participants who achieved ≥5% weight loss, and those who achieved only a small degree of weight loss or remained weight stable. Dose response relationships in a weight-management service have previously been reported for weight loss and pain prevalence but not pain intensity (43, 44). Also, favorable dose–response relationships between the percentage weight loss and improvements in osteoarthritic pain of the knee have been reported in a meta-analysis (61), and in a large diet and exercise intervention (53). The 4% improvement in TUG times was lower than the 9% considered by Claes et al. (60) as a minimal clinical improvement for patients with knee osteoarthritis, but times were within normative limits for adults (62). While some studies have reported improvements in objective measures of physical function with dietary weight-loss interventions (60), others have reported no significant differences (53, 63) which might be related to the magnitude of weight lost.

### 6.1 Limitations

In addressing the complex relationship between obesity and CMP there are several limitations to consider in this study. Firstly, the primary study governed the study design, and as such, the lack of a weight-stable control group limited our interpretation of pain outcomes as a function of time. Further, the predetermined sample restricted our analyses, as less than half of enrolled participants had CMP. Pain outcomes were limited by how pain data were captured. Participants were asked to report any bodily pain in the previous 24 h meaning that some participants with CMP were not captured at baseline if they had a pain-free day preceding assessment. This created difficulty establishing CMP in participants reporting pain of a chronic nature at 3-months that was not present at baseline. Participants also ranked their pain from most to least troublesome, meaning analyses were primarily made from pain at the most troublesome site, rather than being able to assess changes in pain at the same site. Further, VAS was not captured in all participants, being introduced into data collection 3-months post study commencement. Considering the study population were not a pain population, pain experienced by most participants was rated (via VAS) as mild (64) constraining the ability to establish relationships between those with and without CMP.

We did not measure levels of IL-6 in our study, hsCRP is an indicator of IL-6, given CRP’s production in the liver is largely in response to Il-6 (65). Additionally, CRP has more defined reference values than IL-6 (66), making it a potential clinical marker for chronic inflammatory states in musculoskeletal disorders (57).

The scanning limits of the DEXA meant eligibility was restricted to an upper BMI limit of 34.9 kg/m^2^, additionally, history (duration) of overweight/obesity was not captured. The BMI range therefore may not be high enough or weight status not sufficiently long-term to establish inflammatory patterns (9, 67). In our study ~40% of participants had overweight and ~ 60% obesity, and BMI range (27.5–34.9 kg/m^2^) was lower than reported in systematic reviews examining weight-loss interventions for pain, where the average BMIs ranged from ≥25–51 kg/m^2^ (27–29). Finally, most participants achieved at least a 5% weight reduction compared to a smaller weight stable group, potentially impacting the ability to infer statistical significance between these groups.

## 7 Conclusion

Chronic musculoskeletal pain was common in participants with overweight or obesity enrolled in a weight-loss dietary intervention trial. Weight loss, even in small amounts, resulted in a reduction in pain prevalence, severity, and intensity. Improvements in measures of adiposity and functional mobility were observed irrespective of whether participants had CMP at the beginning of the study, indicating that weight loss can be beneficial for both pain-related and general health outcomes.

## Data availability statement

The raw data supporting the conclusions of this article will be made available by the authors, without undue reservation.

## Ethics statement

The studies involving humans were approved by the University of South Australia Human Research Ethics Committee (Application ID: 201436). The studies were conducted in accordance with the local legislation and institutional requirements. The participants provided their written informed consent to participate in this study.

## Author contributions

SW: Data curation, Formal analysis, Investigation, Methodology, Writing – original draft. AC: Conceptualization, Data curation, Funding acquisition, Investigation, Methodology, Project administration, Supervision, Writing – review & editing. SC: Data curation, Methodology, Project administration, Writing – review & editing. KB: Investigation, Methodology, Writing – review & editing, Supervision. CB: Methodology, Writing – review & editing. TS: Methodology, Writing – review & editing. CY: Methodology, Writing – review & editing, Data curation, Project administration. JB: Methodology, Project administration, Writing – review & editing, Conceptualization, Funding acquisition. S-YT: Conceptualization, Funding acquisition, Methodology, Writing – review & editing. GR: Conceptualization, Funding acquisition, Methodology, Writing – review & editing. AH: Conceptualization, Funding acquisition, Methodology, Writing – review & editing, Data curation, Investigation, Project administration, Supervision.
