# Supplementary material for: Effects of weight loss through dietary intervention on pain characteristics, functional mobility, and inflammation in adults with elevated adiposity
Source: Front Nutr. 2024 May 22;11:1274356. doi: 10.3389/fnut.2024.1274356 (PMC11150618; doi:10.3389/fnut.2024.1274356)
Supplement: Supplementary file 1 [file Table_1.docx]

**Supplementary Table S1.** Baseline characteristics for completers (n = 110) by CMP status.

|  | **All participants**  **n=110** | **Participants with CMP**  **n=56** | **Participants without CMP**  **n=54** |
| --- | --- | --- | --- |
| **Women: Men, n (%)^*^** | 77 (70): 33 (30) | 41 (37): 15 (14) | 36 (33): 18 (16) |
| **Age (years)** | 47.9 ± 10.8 | 49.2 ± 10.1 | 46.7 ±10.7 |
| **SEIFA (0, disadvantage - 10, advantage)**^†^**^‡^** | 7.0 ± 4.0 | 8.0 ± 4.0 | 7.0 ± 3.0 |
| **Medications, n (%)^*^**  None  Lipid lowering  Antihypertensive  Anti-anxiety/depression  Hormone Replacement  Analgesic  Reflux  Other (contraceptive etc.) | 55 (50.0)  5 (4.5)  9 (8.2)  15 (13.6)  10 (9.1)  10 (9.1)  5 (4.5)  25 (22.7) | 23 (41.1)  3 (5.4)  5 (8.9)  9 (16.1)  5 (8.9)  7 (12.5)  4 (7.1)  16 (28.6) | 32 (59.3)  2 (3.7)  4 (7.4)  6 (11.1)  5 (9.3)  3 (5.6)  1 (1.8)  9 (16.7) |
| **Supplements, n (%)^*^**  None  Multi/single vitamin/mineral  Omega-3 Fatty acid  Probiotic  Calcium/Vitamin D  Other (curcumin, glucosamine) | 79 (71.8)  17 (15.5)  9 (8.2)  1 (0.9)  13 (11.8)  6 (5.5) | 40 (71.4)  8 (14.3)  6 (10.7)  0 (0.0)  8 (14.3)  4 (7.1) | 39 (72.2)  9 (16.7)  3 (5.6)  1 (1.9)  5 (9.3)  2 (3.7) |
| **Energy Intake (kJ/day)^‡^** | 9111.4 ± 2016.7 | 9334.7 ± 1909.4 | 8875.5 ± 2116.8 |
| **Weight (kg)** | 88.1 ± 11.5 | 88.4 ± 10.8 | 87.8 ± 12.0 |
| **BMI (kg/m^2^)** | 30.7 ± 2.3 | 30.9 ± 2.3 | 30.5 ± 2.3 |
| **Waist Circumference (cm)** | 102.2 ± 9.3 | 102.6 ± 9.4 | 101.8 ± 9.3 |
| **Body composition (DEXA)**  Total Fat Mass (kg)  Total Percent Fat Mass (%)  Total Lean Mass (kg)  Total Percent Lean Mass (%) | 36.2 ± 6.4  42.8 ± 6.0  48.6 ± 8.9  55.4 ± 5.6 | 36.5 ± 6.2  42.9 ± 5.7  48.6 ± 8.3  55.2 ± 5.4 | 35.9 ± 6.5  42.6 ± 6.3  48.7 ± 9.5  55.6 ± 5.9 |
| **Functional mobility**^†^**^‡^**  TUG (sec) | 5.1 ± 0.9 | 5.2 ± 0.9 | 5.1 ± 1.0 |
| **Inflammation**^†§^  hsCRP (mg/L) | 1.8 ± 1.9 | 1.6 ± 2.2 | 1.9 ± 1.7 |
| **Pain reported, n (%)**  Pain not CMP  CMP reported | 75 (68.2)  19 (17.3)  56 (50.9) |  |  |
| **CMP Pain Location**  Lower back  Neck  Thoracic  Shoulder  Hand  Jaw  Hip  Knee  Foot  Lower limb |  | 18 (32.1)  13 (23.2)  4 (7.1)  2 (3.6)  2 (3.6)  1 (1.8)  1 (1.8)  4 (7.1)  8 (14.3)  3 (5.4) |  |
| **AIHW MSK classifications, n (%)**^¶^  No diagnosis  Back pain/problems  Osteo arthritis  Rheumatoid arthritis  Osteoporosis  Other MSK condition (gout, soft tissue etc.)  **MSK fracture or surgery (past 5 years)** |  | 28 (52.8)  8 (15.1)  9 (17.0)  0 (0.0)  0 (0.0)  8 (15.1)  9 (17.0) |  |
| **Pain medication classification, n (%)**^¶^  No medication  Paracetamol  NSAID  Opioid  Antidepressant (for pain)  Anticonvulsant  Supplement (for pain) |  | 30 (56.6)  11 (20.8)  16 (30.2)  1 (1.9)  0 (0.0)  1 (1.9)  1 (1.9) |  |
| **MPQ worst CMP site (0-45),**  (n = 56) |  | 7.8 ± 6.3 |  |
| **VAS worst CMP site (0-10),**  (n = 39) |  | 4.1 ± 2.2 |  |

^*^Relative numbers (n, %). Values are mean ± standard deviation for normally distributed data. ^†^Median ± interquartile range for skewed data. **^‡^**n = 109 (CMP n = 56, no CMP n = 53). ^§^hsCRP n = 99 (CMP n = 49, no CMP n = 50), missing data, n = 6, excluded data for levels ≥ 10 mg/L, n = 5. ^¶^Reported in participants with CMP only, missing data, n = 3.

Abbreviations: AIHW, Australian Institute of Health and Welfare; CMP, chronic musculoskeletal pain; hsCRP, high sensitivity C-reactive protein; DEXA, dual-energy X-ray absorptiometry; IQR, interquartile range; MPQ, McGill Pain Questionnaire; MSK, musculoskeletal; NSAID, non-steroidal anti-inflammatory drugs; SEIFA, Socio-Economic Indices for Areas; SD, standard deviation; TUG, timed up and go; VAS, visual analogue scale.
