# Supplementary material for: Effects of weight loss through dietary intervention on pain characteristics, functional mobility, and inflammation in adults with elevated adiposity
Source: Front Nutr. 2024 May 22;11:1274356. doi: 10.3389/fnut.2024.1274356 (PMC11150618; doi:10.3389/fnut.2024.1274356)
Supplement: Supplementary file 2 [file Table_2.pdf]

Supplementary Material

Table S2. Goodness-of-fit statistics for the mixed effect models

	**Weight (kg)**		**BMI (kg/m^2)**		**WC (cm)**		**Fat mass (kg)**		**% Fat mass**		**Lean mass (kg)**		**% Lean mass**		**TUG (sec)**		**hsCRP (mg/L)**		**Number of pain sites**		**MPQ worst site**		**MPQ matched site**		**VAS worst site**		**VAS matched site**	
	**AIC**	**BIC**	**AIC**	**BIC**	**AIC**	**BIC**	**AIC**	**BIC**	**AIC**	**BIC**	**AIC**	**BIC**	**AIC**	**BIC**	**AIC**	**BIC**	**AIC**	**BIC**	**AIC**	**BIC**	**AIC**	**BIC**	**AIC**	**BIC**	**AIC**	**BIC**	**AIC**	**BIC**
**Models considered for Table 2**																														
Model A	1383	1390	856	862	1310	1317	3759	3775	1011	1017	3574	3580	979	985	452	459	720	726										
Model B	1366	1373	848	855	1294	1300	3712	3718	999	1005	3524	3530	967	974	451	458	709	715										
Model C	1379	1385	854	861	1306	1313	3751	3758	1008	1015	3557	3564	976	982	453	459	718	724										
Model D	1378	1384	853	860	1306	1312	3750	3757	1008	1015	3557	3563	976	982	448	455	719	725										
Model E[*]	1358	1365	845	851	1286	1292	3677	3683	994	1000	3490	3497	962	969	449	455	705	712										
**Models considered for Supplementary Table S3**																														
Model A																			288	293	650	655	573	578	359	364	230[†]	234[†]
Model E[*]																			282	288	637	643	557	562	353[†]	357[†]	225	228
**Models considered for Table 3**																														
Model A			779	786	1275	1281	3724	3731	979	985	3548	3554	948	954	445	451	718	724	287	293	642	647	567	572	358[†]	362[†]	226[†]	229[†]
Model E[*]			769	775	1252	1259	3633	3640	961	968	3465	3471	931	937	443	450	701	708	282	287	629	634	550	555	349[†]	353[†]	220[†]	224[†]

Model Legend

Model A: Age, gender, baseline BMI (for non weight related outcomes)
Model B: Model A plus SEIFA (tertiles)
Model C: Model A plus regular analgesic use (yes/no)
Model D: Model A plus anti-inflammatory/analgesic supplement use (yes/no)
Model E: Model A plus SEIFA (tertiles), regular analgesic use (yes/no), anti-inflammatory/analgesic supplement use (yes/no)

[*]Model E demonstrated lower AIC and BIC values and are presented in the final analyses.
[†]Baseline BMI excluded due to model convergence and validity issues.
Abbeviations: AIC, Akaike information criterion; BIC, Bayesian information criterion; BMI, body mass index;
MPQ, McGill Pain Questionnaire; SEIFA, Socio-economic indices for areas; TUG, timed up and go; VAS, Visual Analgue Scale; WC, waist circumference.
