# Supplementary material for: Effects of weight loss through dietary intervention on pain characteristics, functional mobility, and inflammation in adults with elevated adiposity
Source: Front Nutr. 2024 May 22;11:1274356. doi: 10.3389/fnut.2024.1274356 (PMC11150618; doi:10.3389/fnut.2024.1274356)
Supplement: Supplementary file 3 [file Table_3.docx]

Supplementary Material

**Supplementary Table S3.** Model estimated marginal means (± standard error) and mean difference (95% CI) of pain outcomes by time in the CMP group.

|  | **Baseline** | **3-Months** | **Mean difference (95% CI)** |
| --- | --- | --- | --- |
| **Number of CMP sites,** n=56 | 1.6 ± 0.1 | 0.8 ± 0.1 | -0.8 (-1.1, -0.6) |
| **MPQ worst CMP site (0-45),** n=49 | 7.8 ± 0.8 | 4.6 ± 0.8 | -3.2 (-4.8, -1.6) |
| **VAS worst CMP site (0-10),** n=33^†^ | 4.2 ± 0.4 | 2.4 ± 0.3 | -1.8 (-2.8, -0.9) |
| **MPQ matched CMP site (0-45),** n=46 | 7.4 ± 0.9 | 4.3 ± 0.9 | -3.1 (-4.8, -1.4) |
| **VAS matched CMP site (0-10**), n=27 | 4.0 ± 0.4 | 1.4 ± 0.4 | -2.6 (-3.6, -1.5) |

Adjusted for age, gender, baseline BMI, SEIFA (tertiles), analgesic medication use (yes/no), and anti-inflammatory/analgesic supplement use (yes/no). ^†^ Baseline BMI excluded due to model’s convergence and validity issues.

Abbreviations: CMP, chronic musculoskeletal pain; CI, Confidence Interval; MPQ, McGill Pain Questionnaire; SEIFA, Socio-Economic Indices for Areas; VAS, visual analogue scale.
