# Supplementary material for: Effects of weight loss through dietary intervention on pain characteristics, functional mobility, and inflammation in adults with elevated adiposity
Source: Front Nutr. 2024 May 22;11:1274356. doi: 10.3389/fnut.2024.1274356 (PMC11150618; doi:10.3389/fnut.2024.1274356)
Supplement: Supplementary file 4 [file Table_4.docx]

Supplementary Material

**Supplementary Table S4.** Model estimated associations between pain change and inflammation change limited to CMP participants.

| **Dependent** | **Independent** | $\hat{\boldsymbol{\beta}}$ | **95% CI** | ***p*-value** | **R^2^** |
| --- | --- | --- | --- | --- | --- |
| **∆ no. of pain sites,**  n=39 | **∆ Inflammation, hsCRP (mg/L)** | 0.132 | -0.100, 0.228 | 0.431 | 0.079 |
| **∆ MPQ worst CMP site (0-45),** n=34 | **∆ Inflammation, hsCRP (mg/L)** | 0.153 | -0.646, 1.580 | 0.398 | 0.097 |
| **∆ VAS worst CMP site (0–10),** n=22 | **∆ Inflammation, hsCRP (mg/L)** | 0.406 | -0.097, 1.349 | 0.085 | 0.228 |
| **∆ MPQ matched CMP site (0-45),** n=32 | **∆ Inflammation, hsCRP (mg/L)** | 0.371 | 0.035, 2.470 | 0.044 | 0.189 |
| **∆ VAS matched CMP site (0–10),** n=19 | **∆ Inflammation, hsCRP (mg/L)** | 0.476 | -0.107, 1.846 | 0.077 | 0.272 |

Adjusted for age, gender, and baseline BMI.

Abbreviations: $\hat{\beta}$, standardized regression coefficient; BMI, body mass index; CI, confidence interval; CMP. chronic musculoskeletal pain; hsCRP, high sensitivity C-reactive protein; MPQ, McGill Pain Questionnaire; VAS, Visual Analogue Scale.
